# Prognostic Value of Pro-Inflammatory Neutrophils and C-Reactive Protein in Cancer Patient With Coronavirus Disease 2019: A Multi-Center, Retrospective Study

**DOI:** 10.3389/fphar.2020.576994

**Published:** 2020-10-22

**Authors:** Bo Zhang, Yuanhang Yu, Shawna M. Hubert, Yue Zhang, Jianhua Lu, Shihua Liu, Fang Xie, Liang Zhao, Xiao Lei, Wei Deng, Jianying Chen, Yunqiao Li

**Affiliations:** ^1^ Department of Thyroid and Breast Surgery, Union Hospital, Tongji Medical College, Huazhong University of Science and Technology, Wuhan, China; ^2^ Department of Thoracic Medical Oncology, Department of Genomic Medicine, The University of Texas, MD Anderson Cancer Center, Houston, TX, United States; ^3^ Department of General Surgery, Chibi People’s Hospital, Chibi, China; ^4^ Department of Infectious Diseases, Zaoyang First People’s Hospital, Zaoyang, China; ^5^ Department of Respiratory Intensive Care Unit, Ezhou Central Hospital, Ezhou, China; ^6^ Department of General Surgery, Huangshi Central Hospital, Huangshi, China; ^7^ Department of General Medicine, Union Hospital, Tongji Medical College, Huazhong University of Science and Technology, Wuhan, China; ^8^ Department of Gastrointestinal Surgery, Union Hospital, Tongji Medical College, Huazhong University of Science and Technology, Wuhan, China; ^9^ Department of Geriatrics, Union Hospital, Tongji Medical College, Huazhong University of Science and Technology, Wuhan, China

**Keywords:** cancer, coronavirus disease 2019, pro-inflammatory, neutrophils, C-reactive protein

## Abstract

**Background: **At present, the epidemic of the novel coronavirus disease 2019 (COVID-19) has quickly engulfed the world. Inflammatory cytokines are associated with the severity and outcomes of patients with COVID-19. However, the prognostic value of pro-inflammatory factors in cancer patients with COVID-19 are unknown.

**Methods:** A multi-center, retrospective, cross-sectional study, based on five designated tertiary hospitals for the treatment of COVID-19 in Hubei Province, China. 112 cancer patients with COVID-19, and 105 COVID-19 patients without cancer were enrolled in the study between January 1st, 2020 and April 30th, 2020. The risk assessment of pro-inflammatory factors for disease severity and clinical adverse outcomes was identified by univariable and multivariable logistic regression models.

**Results:** Of the 112 cancer patients with COVID-19, 40 (35.7%) patients were in critical condition and 18 (16.1%) patients died unfortunately. Univariate and multivariate analysis demonstrated that hemoglobin level and pro-inflammatory neutrophils and C-reactive protein (CRP), can be used as independent factors affecting the severity of COVID-19; Meanwhile, pro-inflammatory neutrophils and CRP can be used as an independent influencing factor for adverse clinical outcome of death. Moreover, the dynamic changes of neutrophils and CRP were also presented, and compared with COVID-19 patients without cancer, cancer patients with COVID-19 showed higher neutrophil counts and CRP levels.

**Conclusion:** In cancer patients with COVID-19, the significant increase in pro-inflammatory neutrophils and CRP indicated a more critical illness and adverse clinical outcome, and pro-inflammatory neutrophils and CRP played a greater adverse role compare with COVID-19 patients without cancer, which may be the cause of critical illness and adverse clinical outcomes of cancer patients with COVID-19.

## Introduction

Since the outbreak of coronavirus disease 2019 (COVID-19) in Wuhan in 2019, the outbreak of COVID-19 has quickly spread to Hubei Province and quickly engulfed China (Zhu et al., 2019; Wu and McGoogan, 2020). Now the epidemic has rapidly spread to the worldwide. As of May 16, 2020, COVID-19 had spread to 188 countries around the world, which caused more than 4.5 million people being infected, and more than 300,000 people died. The most common manifestations of COVID-19 infected patients include fever, cough, fatigue, dyspnea, and radiological evidence of pneumonia (Wang et al., 2020). Its mean incubation period was estimated to be 5.2 days (Li et al., 2020). COVID-19 is directly transmitted through respiratory droplets or indirectly through pollutants and be able to secondary transmit within the family (Lotfi et al., 2020).

While facing the epidemic, cancer is still a major disease. According to reports, there were more than 4.2 million new cancer cases in China in 2015 (Chen et al., 2016), and 1.7 billion new cancer cases in the United States in 2019 (Siegel et al., 2019). To data, studies have reported that during this epidemic, cancer patients are more susceptible to COVID-19 (Liang et al., 2020; Stroppa et al., 2020; Yang F. et al., 2020). However, their research was limited to the study of small samples and solid tumors, lack of laboratory data, and insufficient evidence to support relevant conclusions (Wang and Zhang, 2020; Xia et al., 2020).

Moreover, studies have reported that pro-inflammatory factors such as CRP, interleukin 6, neutrophils, etc. were associated with the severity and prognosis of patients with COVID-19 (Liu et al., 2020; Luo et al., 2020; Moore and June, 2020; Sahu et al., 2020). In patients with COVID-19, high levels of pro-inflammatory factors predicted a poor prognosis (Zeng et al., 2020). However, whether inflammatory factors play the same adverse role in cancer patients with COVID-19 is still unclear.

In this study, we collected medical data of 112 cancer patients from five designated tertiary hospitals for the treatment of COVID-19 in Hubei Province, the region where the COVID-19 epidemic occurred earliest and most severely in China. We aim to analyze the laboratory data and outcomes of patients through retrospective research, and then find out the risk correlation of pro-inflammatory factors related to the clinical severity and outcomes of cancer patients with COVID-19. Deepen the understanding of the impact of COVID-19 on cancer patients, demonstrate the adverse role of pro-inflammatory factors in cancer patients with COVID-19, and provide help for the treatment of cancer patients with COVID-19.

## Materials and Methods

### Study Design, Setting and Participants

This was a retrospective, cross-sectional, multicenter study, which was performed in five designated tertiary hospitals for the treatment of COVID-19 in Hubei, China. These data were from 112 cancer patients infected with COVID-19, and 105 COVID-19 patients without cancer, who were enrolled from January 1st to April 30th 2020. The clinical outcomes of these patients were monitored up to June 10th, 2020, the final date follow-up, when all patients were discharged. COVID-19 patients without cancer were randomly selected, and their gender and age were matched with cancer patients with COVID-19.

### Data Collection

Clinical retrospective data was retrieved from the medical records, including demographic information, cancer histories, underlying comorbidities, clinical symptoms, chest computed tomographic scans, laboratory data, anti-tumor therapy, and outcomes. All data were reviewed by two physicians respectively to verify data accuracy. The detailed demographic information, underlying comorbidities, clinical symptoms, and disease severity of all patients were recorded or diagnosed on admission. Laboratory tests, including routine blood tests, inflammatory or infection-related biomarkers, cardiac, renal, liver, and coagulation function tests, were recorded at the time of admission, and were monitored at any time during the hospital according to the development of the disease on average every 2 days.

### Ethics Statement

This study was reviewed and approved by Ethics Committee of the Tongji Medical College of Huazhong University of Science and Technology (No. TJ-2020S098). The exemption from informed patients’ consent was approved by the ethics committee because of the rapid spread of the infection and the rapid progression of some cases.

### Definitions

COVID-19 pneumonia was diagnosed according to the updated COVID-19 Diagnostic Criteria, and clinical diagnostic criteria and laboratory nucleic acid detection confirmation criteria by RT-PCR of nasal and/or pharyngeal specimens. The clinical conditions of cancer patients with COVID-19 and COVID-19 patients without cancer were diagnosed according to Guidelines for the Diagnosis and Treatment of Novel Coronavirus (2019-nCoV) Infection by the National Health Commission of the People’s Republic of China (Trial Version 7). The clinical conditions were divided into moderate, severe and critical. Moderate patients showed fever and respiratory symptoms, and imaging examination showed pneumonia. Severe patients had shortness of breath (breathing rate greater than 30 breaths per minute) or oxygen saturation of less than 93% in the resting state, or arterial oxygen partial pressure (PaO2)/inspired oxygen concentration (FiO2) less than 300 mmHg. Critical patients presented with respiratory failure and required mechanical ventilation or shock, or combined with other organ failure and required intensive care. The evaluation indicators of clinical outcomes are survival and death.

### Statistical Methods

We fitted two types of models. For univariate analysis, categorical variables were expressed as frequency rates and percentages and compared by using Pearson’s chi-square, although the Fisher’s exact test was used when the data were limited, and by using Mann-Whitney test. Continuous variables were described using mean, median, and interquartile range (IQR) values and compared by using independent group *t* tests when the data were normally distributed; otherwise, the Mann-Whitney test was used. In addition, in multivariate analysis, binary logistic regression analysis is used for binary classification variables such as survival status; ordinal logistic regression analysis is used for ordered classification variables such as disease severity. All statistical analyses were performed with the SPSS (version 23.0) software. The data of continuous dynamic detection were subjected to the two-way ANOVA and Student t test by utilizing GraphPad Prism 7. Probability less than 0.05 was considered statistically significant and tests were all two-sided.

## Results

### Presenting Demographic, Clinical Characteristics and Outcome

A total of 112 cancers infected with COVID-19, 105 COVID-19 patients without cancer were included in the study (Table 1). Among COVID-19 patients without cancer, the median age was 68 years old (IQR, 57.0–73.5), 54 (54.2%) patients are male, hypertension (46, 43.8%) was the most common comorbidity, and 31 (29.5%) patients with more than two comorbidities. 45 (42.9%) patients developed severe condition, 16 (15.2%) patients developed critical condition, and 4 (3.8%) patients died unfortunately. Among cancer patients with COVID-19 (Table 2), the median age was 62.95 years old (SD, 15.70), 60 (53.6%) patients are male, 22 (19.6%) patients were non-solid tumor patients. Lung cancer (23, 20.5%) was the most frequent type, following by breast cancer (11, 9.8%) and leukemia (11, 9.8%), hypertension (33, 29.5%) was the most common comorbidity, and 27 (24.1%) patients with multiple comorbidities. 12 (10.7%) patients received anti-tumor therapy with chemotherapy within 28 days before diagnosis of COVID-19, 58 (51.8%) patients developed severe condition, 40 (35.1%) patients developed critical condition, and 18 (16.1%) patients died unfortunately. Compared with COVID-19 patients without cancer, cancer patients with COVID-19 were more critical illness and had a higher mortality (Table 1).

**TABLE 1:** Baseline characteristics of COVID-19 patients.

	COVID-19 patients without cancer (*N* = 105), No. (%)	COVID-19 patients with cancer (*N* = 112), No. (%)	*p* value
Age, median (IQR), year	68.0 (57.0–73.5)	65.0 (57.25–72.75)	0.255
Sex			
Female	51 (45.8)	52 (46.4)	0.752
Male	54 (54.2)	60 (53.6)	
Comorbidities			
Number of Comorbidities per patient >2	31 (29.5)	27 (24.1)	0.368
Hypertension	46 (43.8)	33 (29.5)	0.028
Diabetes	33 (31.4)	20 (17.9)	0.02
Cardiovascular disease	20 (19.0)	10 (8.9)	0.031
Chronic lung disease	5 (4.8)	10 (8.9)	0.227
Chronic bronchitis	1 (1.0)	8 (7.1)	0.036
Hepatitis B	3 (2.9)	7 (6.3)	0.386
Common pulmonary infection	3 (2.9)	4 (3.6)	>0.99
Anemia	1 (1.0)	5 (4.5)	0.214
Arrhythmia	3 (2.9)	4 (3.6)	>0.99
Cerebrovascular disease	6 (5.7)	4 (3.6)	0.668
Pleural effusion	0 (0)	4 (3.6)	0.122
Hyperlipidemia	6 (5.7)	3 (2.7)	0.32
Chronic kidney disease	5 (4.8)	3 (2.7)	0.488
Severity of COVID-19			
Mild	0 (0)	0(0)	<0.001
Moderate	44 (41.9)	14 (12.5)	
Severe	45 (42.9)	58 (51.8)	
Critical	16 (15.2)	40 (35.7)	
Clinical outcomes			
Survival	101 (96.2)	94 (83.9)	0.003
Death	4 (3.8)	18 (16.1)	

*P* Value indicate differences between COVID-19 patients without cancer and cancer patients with COVID-19; *p* < 0.05 was considered statistically significant.

**TABLE 2:** Baseline characteristics of cancer patients with COVID-19.

	Total (*N* = 112), No. (%)	Alive (*N* = 94), No. (%)	Death (*N* = 18), No. (%)	p value
Age, median (IQR), year	62.95±15.70	62.85±15.80	63.44±15.66	0.884
Sex				
Female	52 (46.4)	46 (48.9)	6 (33.3)	0.224
Male	60 (53.6)	48 (51.1)	12 (66.7)	
Tumor nature				
Solid tumor	90 (80.4)	76 (80.9)	14 (77.8)	>0.99
Non-Solid tumor	22 (19.6)	18 (19.1)	4 (22.2)	
Tumor diagnosis				
Lung cancer	23 (20.5)	20 (21.3)	3 (16.7)	0.9
Breast cancer	11 (9.8)	11 (11.7)	0 (0)	0.273
Leukemia	11 (9.8)	10 (10.6)	1 (5.6)	0.817
Colon cancer	10 (8.9)	10 (10.6)	0 (0)	0.318
Liver cancer	9 (8.0)	7 (7.4)	2 (11.1)	0.96
Gastric cancer	6 (5.4)	2 (2.1)	4 (22.2)	0.006
Thyroid cancer	7 (6.3)	6 (6.4)	1 (5.6)	>0.99
Cervical cancer	4 (3.6)	4 (4.3)	0 (0)	>0.99
Rectum cancer	4 (3.6)	4 (4.3)	0 (0)	>0.99
Myeloma	5 (4.5)	4 (4.3)	1 (5.6)	>0.99
Lymphoma	4 (3.6)	3 (3.2)	1 (5.6)	0.509
Bladder cancer	5 (4.5)	5 (5.3)	0 (0)	>0.99
Pancreatic cancer	1 (0.9)	0 (0)	1 (5.6)	0.161
Nasopharynx cancer	2 (1.8)	2 (2.1)	0 (0)	>0.99
Ovarian cancer	2 (1.8)	2 (2.1)	0 (0)	>0.99
Myelodysplastic syndrome	2 (1.8)	1 (1.1)	1 (5.6)	0.297
Prostate cancer	2 (1.8)	1 (1.1)	1 (5.6)	0.297
Esophageal cancer	1 (0.9)	1 (1.1)	0 (0)	>0.99
Oral Cancer	1 (0.9)	0 (0)	1 (5.6)	0.161
Meningioma	1 (0.9)	1 (1.1)	0 (0)	>0.99
Osteosarcoma	1 (0.9)	1 (1.1)	0 (0)	>0.99
Laryngeal cancer	1 (0.9)	1 (1.1)	0 (0)	>0.99
Penile cancer	1 (0.9)	1 (1.1)	0 (0)	>0.99
Skin cancer	1 (0.9)	0 (0)	1 (5.6)	0.161
Comorbidities				
Number of Comorbidities per patient >2	27 (24.1)	17 (18.1)	10 (55.6)	0.002
Hypertension	33 (29.5)	29 (30.9)	4 (22.2)	0.462
Common pulmonary infection	4 (3.6)	2 (2.1)	2 (11.1)	0.121
Diabetes	20 (17.9)	19 (20.2)	1 (5.6)	0.249
Chronic lung disease	10 (8.9)	7 (7.4)	3 (16.7)	0.42
Cardiovascular disease	10 (8.9)	7 (7.4)	3 (16.7)	0.42
Chronic bronchitis	8 (7.1)	5 (5.3)	3 (16.7)	0.225
Hepatitis	7 (6.3)	6 (6.4)	1 (5.6)	>0.99
Cerebrovascular disease	4 (3.6)	2 (2.1)	1 (11.1)	0.121
Arrhythmia	4 (3.6)	2 (2.1)	2 (11.1)	0.121
Anemia	5 (4.5)	3 (3.2)	2 (11.1)	0.182
Pleural effusion	4 (3.6)	2 (2.1)	2 (11.1)	0.121
Hypoproteinemia	4 (3.6)	2 (2.1)	2 (11.1)	0.121
Abnormal thyroid function	1 (0.9)	1 (1.1)	0 (0)	>0.99
Gastritis	3 (2.7)	1 (1.1)	2 (11.1)	0.067
Chronic kidney disease	3 (2.7)	1 (1.1)	2 (11.1)	0.067
Cancer treatment (<28 days)				
Chemotherapy	12 (10.7)	7 (7.4)	5 (27.8)	0.032
Radiotherapy	4 (3.6)	2 (2.1)	2 (11.1)	0.121
Surgery	8 (7.1)	6 (6.4)	2 (11.1)	0.83
Targeted therapy	4 (3.6)	3 (3.2)	1 (11.1)	0.509
Severity of COVID-19				
Moderate	14 (12.5)	14 (14.9)	0 (0)	<0.001
Severe	58 (51.8)	58 (61.7)	0 (0)	
Critical	40 (35.7)	22 (13.4)	18 (100.0)	

*P* values a indicate differences between surviving patients and non-surviving patients; *P* < 0.05 was considered statistically significant.

### Laboratory Findings

Relative to COIVD-19 patients without cancer, cancer patients with COVID-19 show a higher risk of critical illness. Therefore, we analyzed the laboratory parameters of cancer patients with COVID-19 on admission (Table 3). The median or mean of common biochemical indicators of cancer patients with COVID-19 was within the normal reference value range, including platelet count, ×109/L (189.00, IQR [127.50–173.50]), white blood cell count, ×109/L (5.88, IQR [4.44–8.57]), neutrophil count, ×109/L (3.95, IQR [2.73–6.04]), monocyte count, ×109/L (0.42, IQR [0.29–0.63]), aspartate aminotransferase, U/L (30.00, IQR [20.00–47.50]), alanine aminotransferase, U/L (25.00, IQR [18.00–40.00]), alkaline phosphatase, U/L, (77.00, IQR [66.00–114.00]), creatinine, μmol/L (64.65, IQR [53.95–79.83]),creatine kinase, U/L, (63.00, IQR [41.00–93.00]), Lactate dehydrogenase, U/L (230.00, IQR [187.00–353.00]), CK-MB, ng/ml (0.70, IQR [0.40–1.38]), and troponin I, ng/L (5.05, IQR [2.20–12.88]). The erythrocyte count, ×1012/L (3.77, IQR [3.21–4.09]), hemoglobin count, g/L (114.00, IQR [102.00–128.00]), and lymphocyte count, ×109/L (0.99, IQR [0.68–1.56]) are all below the lower limit of the reference value. However, the D-dimer, mg/L (0.92, IQR [0.22–2.39]), and C-reactive protein (CRP), mg/L (35.10, IQR [5.68–81.90]) were significantly higher than the upper limit of the reference value interval. Moreover, in the detection of immune-related factors, the median value of IL-6, pg/ml (18.21, IQR [8.07–42.45]) is significantly higher than the upper limit of the reference value interval, and the median or mean value of the remaining factors are within the normal reference value interval, including TNF-α, pg/ml (2.62, IQR [2.02–3.52]), IL-4, pg/ml (2.26, IQR [1.59–3.32]), IL-2, pg/ml (2.69, IQR [2.37–3.41]), IL-10, pg/ml (4.17, IQR [2.78–5.56]), IFN-γ, pg/ml (2.15, IQR [1.57–3.27]), CD4/CD8 ratio, (1.70, IQR [1.04–2.37). Like the laboratory parameters of cancer patients with COVID-19, the median expression levels of CRP, mg/L (10.95, IQR [1.55–43.18]), and IL-6, pg/ml (12.30, IQR [5.48–28.22]) in COVID-19 patients without cancer were significantly higher than the upper limit of the normal reference range. Relative to the normal reference value range and COIVD-19 patients without cancer, cancer patients with COVID-19 showed a lower erythrocyte count, ×10^9^/L (3.88, IQR [3.21–4.09]) and hemoglobin level, ×10^9^/L (114.00, IQR [102.00–273.50]), a higher neutrophil count, ×10^9^/L (3.95, IQR [2.73–6.04]) and higher levels of CRP, mg/L (35.10, IQR [5.68–81.90]).

**TABLE 3:** Laboratory parameters of COVID-19 patients.

Laboratory parameters	Normal range	COVID-19 patients without cancer (*N* = 105), Median (IQR)	Cancer patients with COVID-19 (*N* = 112), Median (IQR)	*p* value
Erythrocyte count, ×10^12^/L	4.3–5.8	3.96 (3.56–4.29)	3.77 (3.21–4.09)	0.008
Hemoglobin count, g/L	130–175	123.00 (114.00–136.00)	114.00 (102.00–128.00)	0.001
Platelet count, ×10^9^/L	125–350	199.00 (143.00–257.00)	189.00 (127.50–273.50)	0.605
White blood cell count, ×10^9^/L	3.5–9.5	4.99 (4.05–7.00)	5.88 (4.44–8.57)	0.069
Lymphocyte count, ×10^9^/L	1.1–3.2	1.14 (0.82–1.61)	0.99 (0.68–1.56)	0.116
Neutrophil count, ×10^9^/L	1.8–6.3	3.20 (2.42–4.75)	3.95 (2.73–6.04)	0.038
Monocyte count, ×10^9^/L	0.1–0.6	0.48 (0.35–0.66)	0.42 (0.29–0.63)	0.12
Aspartate aminotransferase, U/L	8–40	26.50 (21.00–39.75)	30.00 (20.00–47.50)	0.455
Alanine aminotransferase, U/L	5–40	25.50 (19.00–40.75)	25.00 (18.00–40.00)	0.794
Alkaline phosphatase, U/L	40–150	67.00 (52.25–82.00)	77.00 (62.00–114.00)	0.001
Creatinine, μmol/L	44–133	75.00 (61.18–92.00)	64.65 (53.95–79.83)	0.001
Creatine kinase, U/L	38–174	78.00 (51.00–140.25)	63.00 (41.00–93.00)	0.022
Lactate dehydrogenase, U/L	109–245	229.50 (173.25–291.75)	230.00 (187.00–353.00)	0.187
CK-MB, ng/ml	<6.6	0.70 (0.40–1.20)	0.70 (0.40–1.38)	0.5
Troponin I, ng/L	<26.2	4.65 (1.60–10.25)	5.05 (2.20–12.88)	0.687
Fibrinogen, g/L	2.0–4.0	4.64±1.33	4.30±1.45	0.081^a^
D-dimer, mg/L	<0.50	0.70 (0.33–1.59)	0.92 (0.22–2.39)	0.988
Procalcitonin, μg/L	<0.5	0.04 (0.02–0.88)	0.14 (0.03–0.54)	0.001
CRP, mg/L	<8.00	10.95 (1.55–42.18)	35.10 (5.68–81.90)	0.001
IL-6, pg/ml	0.10–2.90	12.30 (5.48–28.22)	18.21 (8.07–42.45)	0.484
TNF-α, pg/ml	0.10–23.00	2.42 (2.06–3.91)	2.62 (2.02–3.52)	0.874
IL-4, pg/ml	0.10–3.20	2.21 (1.67–3.21)	2.26 (1.59–3.32)	0.784
IL-2, pg/ml	0.10–4.10	2.70 (2.47–3.12)	2.69 (2.37–3.41)	0.471
IL-10, pg/ml	0.10–5.00	3.81 (3.11–5.63)	4.17 (2.78–5.65)	0.989
IFN-γ, pg/ml	0.10–18.00	2.39 (1.86–3.02)	2.15 (1.57–3.27)	0.192
CD4/CD8 ratio, %	0.41–2.72	1.87 (1.40–2.80)	1.70 (1.04–2.37)	0.052

IQR, interquartile range; CRP, C-Reactive Protein; CKMB, Creatine Kinase-MB. P Value indicate differences between COVID-19 patients without cancer and cancer patients with COVID-19; *P* < 0.05 was considered statistically significant.

a Indicates that the SK normality test follows the normal distribution.

### Independent Factors for the Severity of Coronavirus Disease 2019

Further, we analyzed the correlation between inflammatory factors in laboratory parameters and the severity of COVID-19. Univariate analysis showed that the erythrocyte count, hemoglobin count, neutrophil count, lactate dehydrogenase, CK-MB, troponin I, procalcitonin, CRP, IL-6, and IL-10 may be factors that affect the severity of COVID-19 (Table 4). Compared with non-critically condition patients, critically illness patients showed lower erythrocyte count and hemoglobin count, higher neutrophil count and higher levels of LDH, CK-MB, troponin I, procalcitonin, CRP, IL-6, and IL-10. Moreover, Multivariate analysis showed that hemoglobin level (OR, 0.89; 95% CI, 0.81–0.98; *p* = 0.022), neutrophil counts (OR, 1.80; 95% CI, 1.09–2.97; *p* = 0.021), and CRP (OR, 1.04; 95% CI, 1.001–1.071; *p* = 0.043) levels were found to be independent predictors of COVID-19 severity (Table 5). Lower hemoglobin level, higher neutrophil counts, and higher CRP levels were predictive of critical condition. Meanwhile, among COVID-19 patients without cancer, univariate analysis showed that red blood cell count, white blood cell count, lymphocyte count, neutrophil count, lactate dehydrogenase, CK-MB, troponin I, fibrinogen, D-dimer, IL-6, TNF-α, IL-4, IL-2, and CD4/CD8 ratio may be factors affecting the severity of COVID-19 (Supplementary Table S1).

**TABLE 4:** Univariate analysis of the correlation between laboratory parameters and severity of cancer patients with COVID-19.

Laboratory parameters	Moderate (*N* = 14)	Severe (*N* = 58)	Critical (*N* = 40)	*p* value
Erythrocyte count, ×1012/L	3.99 ± 0.55	3.88 ± 0.60	3.12 ± 0.94	<0.001^a^
Hemoglobin count, g/L	123.93 ± 12.06	121.44 ± 17.69	93.00 ± 27.42	<0.001^a^
Platelet count, ×109/L	213.79 ± 70.00	219.29 ± 91.78	181.80 ± 134.17	0.235^a^
White blood cell count, ×109/L	4.39 (3.92–6.32)	5.88 (4.80–8.20)	6.49 (4.67–10.70)	0.083^b^
Lymphocyte count, ×109/L	1.32 ± 0.44	1.41 ± 1.73	7.09 ± 27.18	0.228^b^
Neutrophil count, ×109/L	2.71 (2.17–4.06)	3.95 (2.91–5.83)	4.88 (2.71–8.28)	0.031^b^
Monocyte count, ×109/L	0.44 ± 0.17	0.53 ± 0.27	0.75 ± 2.24	0.668^a^
Aspartate aminotransferase, U/L	27.87 ± 9.68	38.40 ± 26.57	65.76 ± 104.82	0.076^a^
Alanine aminotransferase, U/L	29.38 ± 14.93	35.64 ± 24.31	36.49 ± 38.48	0.73^a^
Alkaline phosphatase, U/L	66.79 ± 23.33	94.98 ± 63.76	245.43 ± 565.63	0.078^a^
Creatinine, μmol/L	62.89 ± 13.89	85.40 ± 107.09	79.92 ± 54.12	0.665^a^
Creatine kinase, U/L	64.00 (48.50–92.15)	62.00 (40.00–87.00)	63.00 (30.00–108.00)	0.903^b^
Lactate dehydrogenase, U/L	193.22 ± 40.96	260.58 ± 107.11	451.86 ± 447.58	0.001^a^
CK-MB, ng/ml	0.83 ± 0.51	0.76 ± 0.52	2.18 ± 3.02	0.004^a^
Troponin I, ng/L	2.53 ± 1.91	8.83 ± 15.55	38.97 ± 93.84	0.041^a^
Fibrinogen, g/L	3.56 ± 1.06	4.45 ± 1.41	4.35 ± 1.58	0.123^a^
D-dimer, mg/L	0.53 ± 0.47	1.67 ± 2.26	3.75 ± 4.96	0.003^a^
Procalcitonin, μg/L	0.04 (0.01–0.13)	0.06 (0.03–0.25)	0.60 (0.22–1.40)	<0.001^b^
CRP, mg/L	0.78 (0.28–11.68)	24.10 (4.90–55.95)	82.33 (44.81–124.50)	<0.001^b^
IL-6, pg/ml	6.42 (3.81–11.00)	10.05 (4.45–29.51)	32.79 (10.51–53.41)	<0.001^b^
TNF-α, pg/ml	4.62 ± 3.51	6.05 ± 11.51	3.19 ± 3.66	0.399^a^
IL-4, pg/ml	2.27 ± 0.78	3.50 ± 5.35	2.46 ± 1.45	0.486^a^
IL-2, pg/ml	2.67 ± 0.40	3.03 ± 0.90	2.86 ± 1.23	0.591^a^
IL-10, pg/ml	3.30 ± 0.83	4.07 ± 1.73	6.19 ± 3.97	0.003^a^
IFN-γ, pg/ml	2.09 ± 0.66	2.47 ± 1.04	3.08 ± 3.73	0.471^a^
CD3 ratio, %	81.06 ± 6.98	66.42 ± 20.46	70.79 ± 12.53	0.082^a^
CD4 ratio, %	50.87 ± 4.41	39.62 ± 14.47	37.40 ± 14.69	0.06^a^
CD8 ratio, %	27.04 ± 4.93	23.69 ± 12.91	28.50 ± 10.96	0.253^a^
CD4/CD8 ratio, %	1.93 ± 0.34	2.08 ± 1.15	1.53 ± 0.96	0.108^a^

CRP, C-Reactive Protein; CKMB, Creatine Kinase-MB; *p* < 0.05 was considered statistically significant.

a Indicates that the SK normality test follows the normal distribution, and expressed as mean ± standard deviation.

b Indicates that the SK normality test follows a non-normal distribution, and expressed as median (interquartile range).

**TABLE 5:** Multivariate analysis of the correlation between laboratory parameters and severity of cancer patients with COVID-19.

Laboratory parameters	Regression coefficients	OR	*p* value	95% CI
Erythrocyte count, ×10^12^/L	0.682	1.98	0.337	0.20–19.85
Hemoglobin count, g/L	−0.116	0.89	0.022	0.81–0.98
Neutrophil count, ×10^9^/L	0.589	1.80	0.021	1.09–2.97
Lactate dehydrogenase, U/L	0.003	1.00	0.224	0.998–1.007
CK-MB, ng/ml	0.462	1.59	0.595	0.29–8.71
Troponin I, ng/L	0.024	1.02	0.133	0.99–1.06
D-dimer, mg/L	−0.172	0.84	0.456	0.53–1.32
Procalcitonin, μg/L	0.034	1.03	0.831	0.76–1.41
CRP, mg/L	0.035	1.04	0.043	1.001–1.071
IL-6, pg/ml	0.008	1.01	0.405	0.99–1.025
IL-10, pg/ml	0.299	1.35	0.344	0.73–2.50

CRP, C-Reactive Protein; CKMB, Creatine Kinase-MB; OR, Odd Ratio; *p* < 0.05 was considered statistically significant.

### Independent Factors for the Adverse Clinical Outcome of Coronavirus Disease 2019

Meanwhile, we analyzed the correlation between inflammatory factors in laboratory parameters and the adverse clinical outcomes of COVID-19. Univariate analysis showed that erythrocyte count, hemoglobin level, neutrophil counts, LDH, procalcitonin, CRP, and IL-6 may be factors that affect the adverse clinical outcome of COVID-19 (Table 6). Compared to surviving patients, non-surviving patients also showed lower erythrocyte count and hemoglobin level, higher neutrophil counts, and higher levels of LDH, procalcitonin, CRP, and IL-6. Multivariate analysis showed that neutrophil counts (OR, 1.286; 95% CI, 1.007–1.536; p = 0.005) and CRP (OR, 1.047; 95% CI, 1.016–1.079; *p* = 0.003) levels were found to be independent predictors of adverse clinical outcomes of COVID-19 (Table 7). Higher neutrophil counts and higher levels of CRP were predictive of adverse clinical outcomes.

**TABLE 6:** Univariate analysis of the correlation between laboratory parameters and adverse clinical outcome of cancer patients with COVID-19.

Laboratory parameters	Alive (*N* = 94)	Death (*N* = 18)	*p* value
Erythrocyte count, ×10^12^/L	3.69 ± 0.77	3.20 ± 0.99	0.02^a^
Hemoglobin count, g/L	113.63 ± 23.66	99.67 ± 31.06	0.033^a^
Platelet count, ×10^9^/L	210.19 ± 102.75	177.72 ± 130.28	0.245^a^
White blood cell count, ×10^9^/L	5.53 (4.30–7.85)	8.94 (5.94–12.26)	0.003^b^
Lymphocyte count, ×10^9^/L	1.03 (0.79–1.57)	0.86 (0.51–1.39)	0.134^b^
Neutrophil count, ×10^9^/L	3.69 (2.71–5.63)	7.40 (3.66–10.95)	0.003^b^
Monocyte count, ×10^9^/L	0.42 (0.31–0.62)	0.41 (0.20–0.65)	0.542^b^
Aspartate aminotransferase, U/L	29.00 (20.00–45.00)	35.50 (21.25–76.00)	0.428^b^
Alanine aminotransferase, U/L	25.00 (18.00–37.00)	23.50 (15.50–44.25)	0.886^b^
Alkaline phosphatase, U/L	74.00 (61.00–104.00)	103.00 (76.00–288.50)	0.01^b^
Creatinine, μmol/L	65.00 (53.68–79.48)	64.10 (54.18–106.68)	0.556^b^
Creatine kinase, U/L	63.00 (40.50–88.50)	64.50 (42.25–170.25)	0.608^b^
Lactate dehydrogenase, U/L	222.50 (183.78–298.25)	460.55 (223.75–639.25)	0.001^b^
CK-MB, ng/ml	0.70 (0.40–1.10)	1.35 (0.58–2.52)	0.016^b^
Troponin I, ng/L	3.80 (1.90–10.25)	14.10 (6.30–39.38)	0.003^b^
Fibrinogen, g/l	4.28 ± 1.43	4.38 ± 1.62	0.788^a^
D-dimer, mg/L	0.90 (0.24–1.84)	3.18 (0.01–6.57)	0.324^b^
Procalcitonin, μg/L	0.09 (0.03–0.30)	0.61 (0.36–2.52)	<0.001^b^
CRP, mg/L	24.10 (3.79–61.60)	93.02 (77.45–182.25)	<0.001^b^
IL-6, pg/ml	10.10 (5.42–32.68)	39.18 (24.70–89.36)	0.001^b^
TNF-α, pg/ml	2.69 (2.10–3.58)	2.26 (1.50–2.84)	0.09^b^
IL-4, pg/ml	2.27 (1.63–3.34)	2.10 (1.40–2.77)	0.405^b^
IL-2, pg/ml	2.69 (2.39–3.53)	2.59 (2.18–3.27)	0.521^b^
IL-10, pg/ml	4.01 (2.70–5.38)	5.34 (3.32–8.98)	0.09^b^
IFN-γ, pg/ml	2.19 (1.63–3.29)	1.78 (1.38–2.83)	0.129^b^
CD3 ratio, %	69.90 ± 17.82	68.19 ± 14.08	0.764^a^
CD4 ratio, %	40.54 ± 14.13	37.15 ± 15.28	0.471^a^
CD8 ratio, %	26.27 ± 12.16	23.38 ± 8.64	0.453^a^
CD4/CD8 ratio, %	1.88 ± 1.03	1.75 ± 1.16	0.705^a^

CRP, C-Reactive Protein; CKMB, Creatine Kinase-MB; *P* < 0.05 was considered statistically significant.

a Indicates that the SK normality test follows the normal distribution, and expressed as mean ± standard deviation.

b Indicates that the SK normality test follows a non-normal distribution, and expressed as median (interquartile range).

**TABLE 7:** Multivariate analysis of the correlation between laboratory parameters and clinical adverse outcomes in cancer patients with COVID-19.

Clinical factor	Regression coefficients	OR	*p* value	95% CI
Erythrocyte count, ×10^12^/L	−0.86	0.423	0.704	0.005–35.822
Hemoglobin count, g/L	0.33	1.034	0.656	0.894–1.196
Neutrophil count, ×10^9^/L	0.252	1.286	0.005	1.007–1.536
Lactate dehydrogenase, U/L	0.002	1.002	0.104	1.000–1.004
Procalcitonin, μg/L	−0.056	0.945	0.319	0.847–1.056
CRP, mg/L	0.046	1.047	0.003	1.016–1.079
IL-6, pg/ml	0.011	1.011	0.153	0.996–1.026

CRP, C-Reactive Protein; *P* < 0.05 was considered statistically significant.

### Dynamic Changes of Pro-Inflammatory Neutrophils and C-Reactive Protein

**FIGURE 1:**
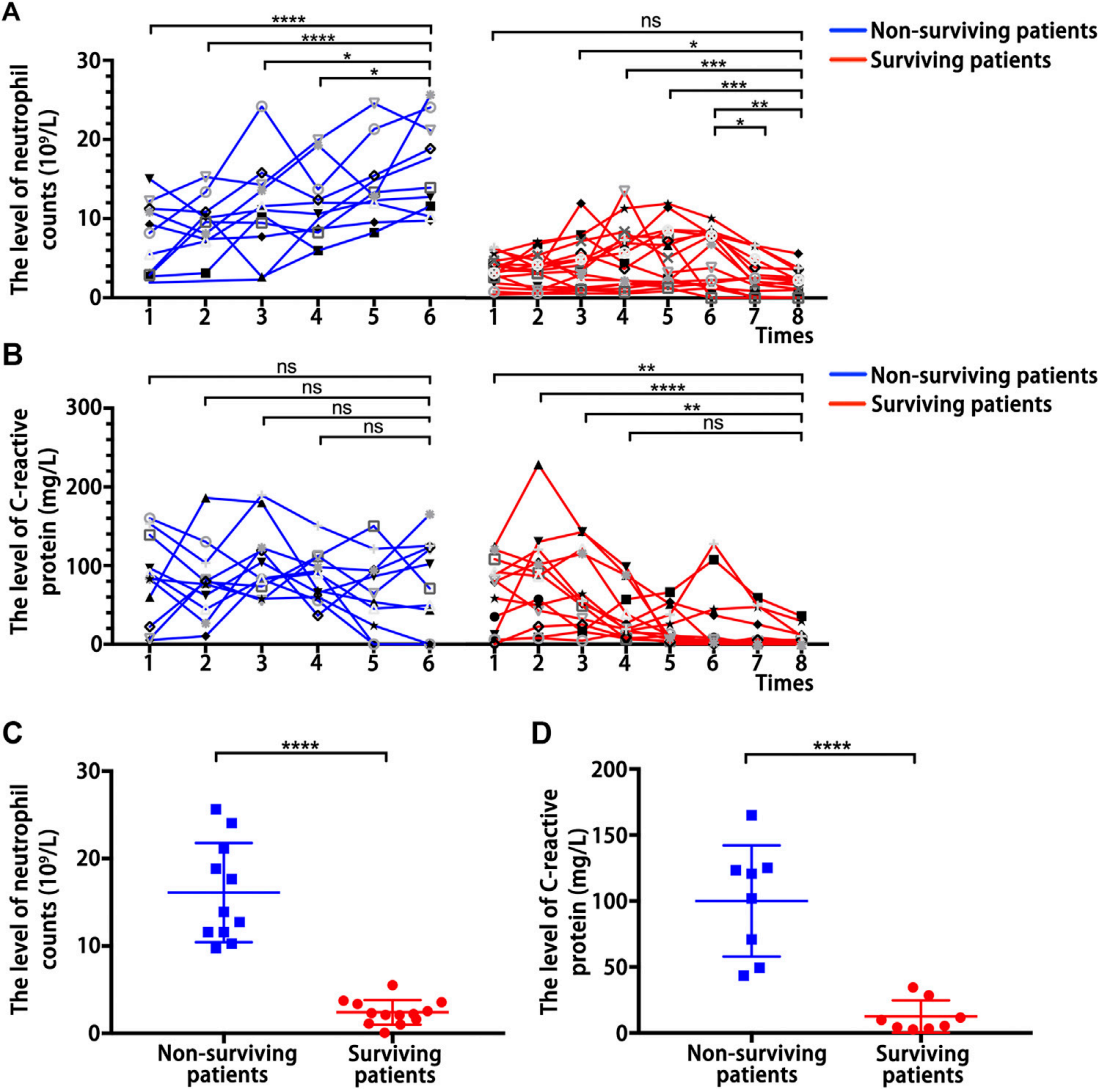
Dynamic changes of inflammation-related indicators. **(A)** Dynamic changes of neutrophil counts in surviving patients and non-surviving patients with COVID-19. **(B)** Dynamic changes of C-reactive protein levels in surviving patients and non-surviving patients with COVID-19. **(C)** Neutrophil counts detected at follow-up endpoints in surviving and non-surviving patients. **(D)** C-reactive protein level detected at follow-up endpoints in surviving and non-surviving patients. *P* values were determined by two-tailed *t*-test, ns, not significant; ^*^ *p* < 0.05; ^**^ *P* < 0.01; ^**^ *P* < 0.01; ^****^ *p* <0.0001.

The above results indicated that the pro-inflammatory neutrophils and CRP are independent predictors of COVID-19 severity and clinical adverse outcomes. Therefore, we analyzed the continuous dynamic changes of neutrophil counts and CRP levels in surviving and non-surviving patients in critical condition. This dynamic process of change was from admission to discharge or death, and according to the development of the disease, blood samples were collected at an average interval of 2 days for testing. The neutrophil counts showed an overall higher level and an upward trend in the dynamic changes of non-surviving patients, but it showed an overall lower level and a downward trend in the dynamic changes of surviving patients (Figure 1A). Meanwhile, CRP showed an overall higher level in the dynamic changes of non-surviving patients, but showed an overall lower level and a downward trend in the dynamic changes of surviving patients (Figure 1B). Moreover, the neutrophil counts (Figure 1C) and CRP (Figure 1D) levels of non-surviving patients at follow-up endpoints were significantly higher than those of surviving patients. These further identified the inflammatory cytokines neutrophils and CRP as predictors of the adverse clinical outcome of COVID-19.

### Clinical Factors of Severity and Adverse Clinical Outcomes

Studies have reported that cancer patients with non-solid tumors, comorbidities and chemotherapy after infection with COVID-19 are more critically ill or have a higher mortality rate. Therefore, we analyzed the correlation of clinical factors other than laboratory parameters with COVID-19 severity and adverse clinical outcomes in 112 cancer patients with COVID-19 to verify the reliability of our data. The results showed that patients with non-solid tumor cancer and myeloma were more critical illness after infection with COVID-19 (Supplementary Figures S1A,B). Patients with thyroid cancer were milder after infection with COVID-19 (Supplementary Figure S1C). Cancer patients with anemia, pleural effusion, multiple complications, and chemotherapy were more critical illness after infection with COVID-19 (Supplementary Figures S1D–F). Moreover, gastric cancer patients, cancer patients with multiple comorbidities and chemotherapy had a higher mortality rate after infection with COVID-19 (Supplementary Figures S1H–J).

## Discussion

In this study, we retrospectively analyzed the laboratory data and clinical outcomes of 112 cancer patients with COVID-19 and compared 105 patients with COVID-19 patients without cancer as controls. Our data demonstrated that although univariate analysis indicate that the levels of pro-inflammatory neutrophils, procalcitonin, CRP, and IL-6 are associated with the severity and adverse clinical outcomes of COVID-19, multivariate analysis indicate that only neutrophils and CRP can be used as independent predictors of disease severity and adverse clinical outcomes. Moreover, the dynamic changes of pro-inflammatory neutrophils and CRP indicated that the neutrophils count and the level of CRP in non-surviving patients remain at an overall higher level and gradually increase, and compared with COVID-19 patients without cancer, cancer patients with COVID-19 showed a higher neutrophil count and higher levels of CRP, meanwhile showed more critical illness and higher mortality rate.

Meanwhile, studies have reported that patients infected with COVID-19 show increased levels of pro-inflammatory neutrophils, procalcitonin, CRP and IL-6 (Chen et al., 2020; Zeng et al., 2020). Moreover, studies have reported that the neutrophil count, IL-6 and CRP can be used as predictors of clinical outcome and disease severity (Liu et al., 2020; Luo et al., 2020; Qin et al., 2020). Recently, some studies have shown that cancer patients with COVID-19 have higher level of CRP, and higher neutrophil counts, D-dimer and LDH are associated with poor prognosis (Mehta et al., 2020; Zhang et al., 2020). Our data also demonstrated that the expression of pro-inflammatory neutrophils and CRP was significantly increased in cancer patients with COVID-19, and further through multivariate regression analysis showed that they can be used as independent predictors of the severity and the clinical adverse outcome of COVID-19.

In addition, many studies have reported that cancer patients become more severe and have a poor prognosis after being infected with COVID-19 (Dai et al., 2020; Mehta et al., 2020). Recent research on cancer patients with COVID-19 had shown that non-surviving patients exhibit higher neutrophil counts and CRP levels (Yang K. et al., 2020). However, the correlation between the dynamic changes of cytokine levels and the prognosis of patients during disease has not been studied. Therefore, while discovering the same results, we further analyzed the dynamic changes of pro-inflammatory neutrophils and CRP, and further verified that the pro-inflammatory neutrophils and CRP were adverse effects in cancer patients with COVID-19.

Meanwhile, studies have reported that inflammation promotes tumorigenesis and development at all stages, and tumors can also promote the production of inflammatory cells and inflammatory mediators (Greten and Grivennikov, 2019). Inflammation could activate neutrophils and their extracellular trap formation to promote tumor development (Albrengues et al., 2018). Tumor development and progression can also induce inflammation. Neutrophils and CRP are inflammatory marker. Elevated levels of neutrophil counts and CRP may also play a causal role in the pathogenesis of cancer (Allin and Nordestgaard, 2011). It has been reported that in patients infected with COVID-19, some functionally activated neutrophils can form neutrophil extracellular traps, may contributing to organ damage and mortality (Barnes et al., 2020). Meanwhile, excessive neutrophil extracellular traps formation can trigger a cascade of inflammatory reactions that promotes cancer cell metastasis, destroys surrounding tissues, and results in permanent organ damage (Jorch and Kubes, 2017; Papayannopoulos, 2018). Therefore, we believe that pro-inflammatory neutrophils, that is, functionally activated neutrophils, have a powerful function of promoting inflammation and causing organ damage. Moreover, COVID-19 as an acute inflammatory disease. Due to coronavirus infection, monocytes and macrophages secrete large amounts of inflammatory cytokines and inflammatory mediators such as CRP and IL-6. This can trigger cytokine release syndrome, which causes ARDS to cause death (Moore and June, 2020).

Therefore, higher neutrophil counts and CRP levels in cancer patients with COVID-19 may be the result of the combined effect of cancer and COVID-19, and lead to mutual promotion, and may also be the key to triggering a “cytokine storm” and leading to critical illness and mortality. Pro-inflammatory neutrophils and CRP may be one of the factors that make the cancer patients more serious and have a poor prognosis after being infected with COVID-19.

Although we demonstrated the adverse roles of pro-inflammatory neutrophils and CRP in cancer patients with COVID-19, our research still had several limitations. First, this was a retrospective study, and the number of study cases is somewhat small. There were also deficiencies in the number of medical records in the control group. Second, we did not consider enough risk factors to establish a risk assessment model and have not fully considered the unmeasured confounding factors. In the end, the mechanism by which pro-inflammatory neutrophils and CRP aggravate the disease of COVID-19 and cause poor prognosis remains to be further investigated.

In summary, we presented some clinical features, laboratory data and outcomes of cancer patients after being infected with COVID-19. Pro-inflammatory neutrophils and CRP can be used as independent predictors of the severity and the adverse clinical outcomes of COVID-19. Moreover, compared with COVID-19 patients without cancer, cancer patients with COVID-19 have higher neutrophil counts and CRP levels. Therefore, pro-inflammatory neutrophils and CRP play a greater adverse role in patients with COVID-19 cancer, as a mediator to accelerate the interaction of cancer and COVID-19, and promote the occurrence of “cytokine storm”, which may be COVID-19 causes of critical illness and adverse clinical outcomes in cancer patients with COVID-19. During the outbreak of COVID-19, wo need to be more alert to the levels of pro-inflammatory neutrophils and CRP after cancer patients infected with COVID-19, so as to better give corresponding treatment measures.

## Data Availability Statement

The datasets presented in this article are not readily available because the application of the dataset requires the consent of the corresponding author. Requests to access the datasets should be directed to Jianying Chen, bobytail@sina.com.

## Ethics Statement

The studies involving human participants were reviewed and approved by Ethics Committee of the Tongji Medical College of Huazhong University of Science and Technology (No. TJ-2020S098). Written informed consent for participation was not provided by the participants’ legal guardians/next of kin because the exemption from informed patients’ consent was approved by the ethics committee because of the rapid spread of the infection and the rapid progression of some cases.

## Author Contributions

YL and JC designed the study and take responsibility for the integrity of the data and the accuracy of the data analysis. BZ and YY interpreted the results and wrote the manuscript. YY performed the analysis. SH substantially revised it. YZ, JL, SL, FX, LZ, XL, and WD collected and analyzed the data. All authors read and approved the final manuscript.

## Funding

This work was supported by the National Natural Science Foundation of China [81773197 to BZ]; Nature Science Foundation of Hubei Province [2017CFB412 to JC, 2017CFB633 to YL]; and the joint research foundation of Union Hospital in 2016.

## Conflict of Interest

The authors declare that the research was conducted in the absence of any commercial or financial relationships that could be construed as a potential conflict of interest.

## Abbreviations

**Abbreviations:** CK-MB, creatine kinase‐MB; CRP, C-reactive protein; IL, interleukin; LDH, lactate dehydrogenase.
